# Vascular Disease and Diabetes

**DOI:** 10.3400/avd.ra.24-00010

**Published:** 2024-03-13

**Authors:** Hiroyoshi Komai

**Affiliations:** 1 Department of Vascular Surgery, Kansai Medical University Medical Center, Osaka, Osaka, Japan

**Keywords:** vascular disease, diabetes, atherosclerosis obliterans, diabetic foot

## Abstract

The most important vascular lesion associated with diabetes is arteriosclerosis obliterans (ASO). Differential diagnosis from diabetic foot lesions that produce neurogenic ulcers is important, and the presence of ischemia must be diagnosed as soon as possible. It has been reported that diabetes makes ASO more severe and often leads to lower extremity amputation. In addition to the need for appropriate early control of diabetes, vascular surgeons are required to perform immediate revascularization in cases of ulcer and necrosis, and to aggressively use surgical treatment with good long-term prognosis. (This is a translation of Jpn J Vasc Surg 2023; 32: 105–109.)

## Introduction

Diabetes has spread worldwide like a pandemic. In 2015, the estimated number of patients with diabetes worldwide was 415 million, and this number is expected to rise to 642 million by 2024.^1)^ Among the pathological effects of diabetes, three major complications are widely known, namely neuropathy, renal failure, and retinopathy. However, another very important complication is angiopathy, which affects all the arteries in the body. Advanced arteriosclerosis causes organ damage, and in particular, the heart, brain, and lower extremities are organs that are closely related to vascular surgeons. Furthermore, diabetic foot, including neurogenic ulcers and necrosis of the distal parts of the lower extremities, necessitates close medical care by vascular surgeons, although strictly speaking, this is not a vascular disease. This paper provides a commentary about arteriosclerosis obliterans (ASO), which is a typical vascular disease of the lower limbs, and makes reference to diabetic foot as a condition to differentiate from ASO.

## Diabetes and Foot Lesions

Foot lesions caused by diabetes are broadly divided into two types. One type is diabetic foot, which characterized by foot ulcers and necrosis caused by neuropathy and foot deformity but is not necessarily accompanied by impaired blood flow. The other type includes ulcers and necrosis associated with impaired blood flow caused by reduced blood supply in the lower limbs due to diabetes-induced arteriosclerosis. Even in developed countries, the annual incidence of foot lesions caused by diabetes is approximately 2%, and approximately 1% of patients with diabetes undergo lower extremity amputation.^2)^ The treatment approaches for neurogenic and ischemic lesions are completely different, and it is thus important to differentiate between the two; however, in recent years, it has been found that many lesions are neuroischemic, i.e., concurrently neurogenic and ischemic. The 2017 revised version of European guidelines of peripheral arterial disease (PAD) and the Global Vascular Guidelines (GVG), which are global guidelines for severe ischemia, have been created on the premise that there are more patients requiring revascularization than before.

## Diabetes and ASO

In the case of diabetes, hyperglycemia leads to an increase in advanced glycation end-products (AGEs) and promotes oxidative stress and inflammation, which cause vascular damages, including ASO.^3)^ Furthermore, diabetes is associated with thrombogenic tendency, and it has also been reported that the abovementioned AGEs, C-reactive protein, and oxidated low-density lipoproteins facilitate intimal plaque rupture by producing matrix metalloproteinase-9. All of these promote arteriosclerosis progression; therefore, the incidence of ASO is approximately 2–4-fold higher,^4)^ and the incidence of ulcers caused by severe ischemia is also approximately 4-fold greater in patients with type 2 diabetes than that in nondiabetic patients.^5)^ Moreover, the incidence of ASO has been reported to increase by 26% for every 1% increase in hemoglobin A1c (HbA1c) levels.^6)^ According to the data of Japanese cases in the Reduction of Atherothrombosis for Continued Health (REACH) registry, which is a registry study of arteriosclerotic disease, the rate of concurrent diabetes in patients with PAD is 42.1%,^7)^ and in the Japanese Critical Limb Ischemia database (JCLIMB), the largest database of critical limb ischemia (CLI) in Japan, the rate of concurrent diabetes among patients with CLI is as high as 67%.^8)^

The three major complications of diabetes are deeply involved in the aggravation of foot lesions. When neuropathy is present, it is possible that pain at rest and pain caused by ulcers and necrosis, as well as claudication symptoms caused by ischemia, are hidden, thereby delaying their detection. It has been reported that approximately half of patients who developed chronic limb-threatening ischemia did not have a history of claudication.^9)^ Poor vision due to retinopathy also causes a delay in noticing foot abnormalities. Patients with renal failure and those undergoing dialysis have many risk factors for arteriosclerosis and are thus prone to more serious aggravation of lower limb ischemia. Reduced renal function in type 1 and type 2 diabetes greatly contributes to diabetic foot.^10)^ In a meta-analysis, it has been reported that the risk of PAD onset and leg amputation is increased in patients with renal insufficiency requiring dialysis and even in those with early stage renal dysfunction.^11)^ It has also been reported that smoking, a factor in ASO onset, increases the onset of diabetes and that the mutual effect of ASO and diabetes promotes arteriosclerosis even more.^12)^ Cardiovascular complications often make diabetes fatal; however, it has also been reported that when diabetes is concurrent with PAD, the annual cardiovascular death rate roughly doubles from 1.7% to 3.1%,^13)^ making the presence or absence of diabetes a major factor that affects the feet as well as the survival of patients with PAD.

## Diagnosis of ASO in Patients with Diabetes

**Figure 1:**
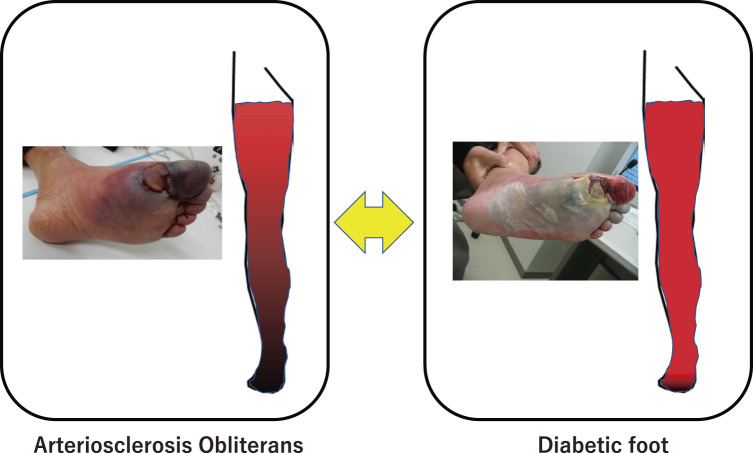
Fig. 1 Differences of blood flow between ASO and diabetic foot lesions. In ASO, blood flow deteriorates toward the periphery, whereas in diabetic foot, blood flow is often preserved even around the ulcer. Despite similar findings, the differential diagnosis between the two is very important. ASO: arteriosclerosis obliterans

It is important to detect lower limb ischemia at an early stage in patients with diabetes. As mentioned above, neuropathy and visual impairment often delay detection by the patient; thus, the involvement of family members and healthcare workers is essential. In patients with foot lesions, the diagnosis of whether the lesion is neurogenic or a complication of ischemia should be made first (**Fig. 1**). The ankle–brachial index (ABI) is a useful tool to screen for PAD; however, in patients with diabetes, calcification of the media due to Mönckeberg’s arteriosclerosis often makes the ABI inaccurate. Therefore, to diagnose lower limb ischemia, the toe–brachial index^14)^ and the skin perfusion pressure measurement^15)^ are more accurate; however, such measures have not yet gained popularity and time is needed to obtain measurements, which hinders their popularization. Recently, we have developed a new technique to diagnose ischemia of the lower limbs that can be performed easily as a bedside examination using percutaneous oxygen saturation measurement.^16)^ In any case, when foot lesions are detected in patients with diabetes, the presence or absence of ischemia, i.e. the presence or absence of ASO, should be diagnosed before simply performing debridement. Accurate diagnosis using enhanced computed tomography and angiography is needed for patients needed revascularization. Contrast medium must be used very cautiously for patients with renal dysfunction caused by diabetes, which is also a reason that treatment becomes inadequate or is delayed. At the same time, the presence or absence of infection must be determined accurately. Patients with diabetes have very poor resistance to infection, and due caution should always be exercised in cases of septicemia resulting from wounds. It is important to suspect an early infection when redness is detected by visual examination, and heat and tenderness are detected by palpation. Methods used for the diagnosis of osteomyelitis include plain X-rays, magnetic resonance imaging, and a probe-to-bone test (considered positive if a probe inserted into an ulcer touches the bone).^17)^

## Treatment of Diabetes-Related Vascular Lesions

**Figure 2:**
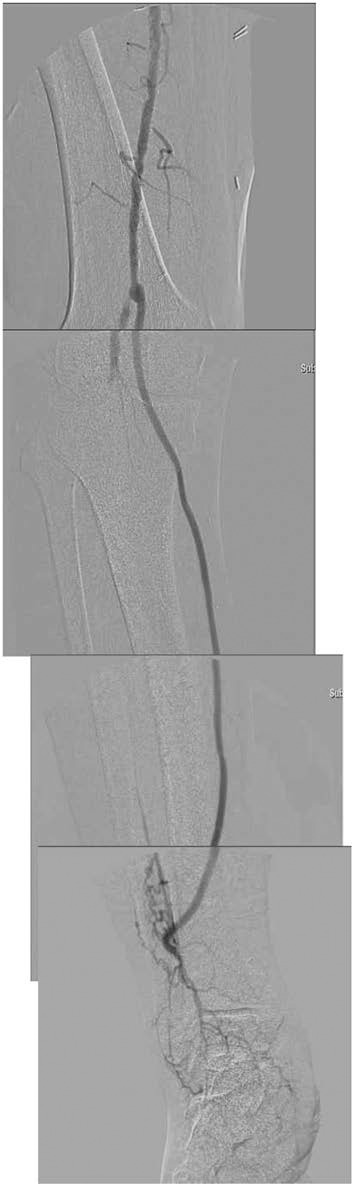
Fig. 2 Angiography of bypass surgery in patients with CLI 10 years after the procedure. High-quality bypass surgery is increasingly required in the future, taking into account not only early but also long-term prognosis in cases with diabetes mellitus. CLI: critical limb ischemia

In patients with diabetes, control of blood sugar levels is essential for preventing vascular disease. It has been recommended to keep HbA1c below 7.0% to prevent microangiopathy and complications.^18)^ However, HbA1c levels to stabilize macroangiopathy, including ASO, remain controversial, and it is said that control of HbA1c and postprandial hyperglycemia is necessary.^19)^ Exercise therapy is an important treatment method for diabetes as well as PAD and helps improve patient survival, cardiovascular risks, and quality of life (QOL).^20)^ In infected wounds, antibiotics that is effective against responsible bacteria should be administered after ensuring blood flow. In neurogenic ulcers, local debridement promotes wound healing, and in patients with concurrent infections, drainage and irrigation by necrotic tissue resection are needed. For wounds with adequate blood flow, negative-pressure wound therapy^21)^ is a very useful device to promote wound healing. In patients with concurrent ischemia, revascularization is essential; however, the strategy to achieve this should be carefully considered to obtain early wound healing.^22)^ While endovascular treatment is noninvasive and can be conducted quickly, restenosis occurs often in tibial lesions commonly seen in patients with diabetes,^23)^ and it is often an inadequate treatment for severely calcified lesions. Arterial bypass restores abundant blood flow immediately and enables early wound healing to be achieved; however, it is invasive and carries a high risk for diabetic patients with many complications. The Global Anatomic Staging System (GLASS) of the GVG and the surgical treatment selection score of the SPINACH study^9)^ can be used to select an appropriate revascularization method. Patients with concurrent diabetes are at high risk, and less invasive endovascular treatment is preferred for mild cases; however, it has been pointed out that for patients who present with claudication symptoms, early endovascular treatment can worsen patient prognosis.^24)^ The BEST-CLI trial, which is a recent multicenter, large-scale, prospective randomized trial,^25)^ has provided data indicating that long-term prognosis yields better results in the surgical treatment group. Treatment should ultimately be determined by comprehensively considering the individual patient’s condition, social adaptation, and level of the surgeon’s skill; however, vascular surgeons who have all treatment options should proactively seek good-quality surgical treatment (**Fig. 2**).

## Issues in the Treatment of Vascular Lesions in Patients with Diabetes

**Figure 3:**
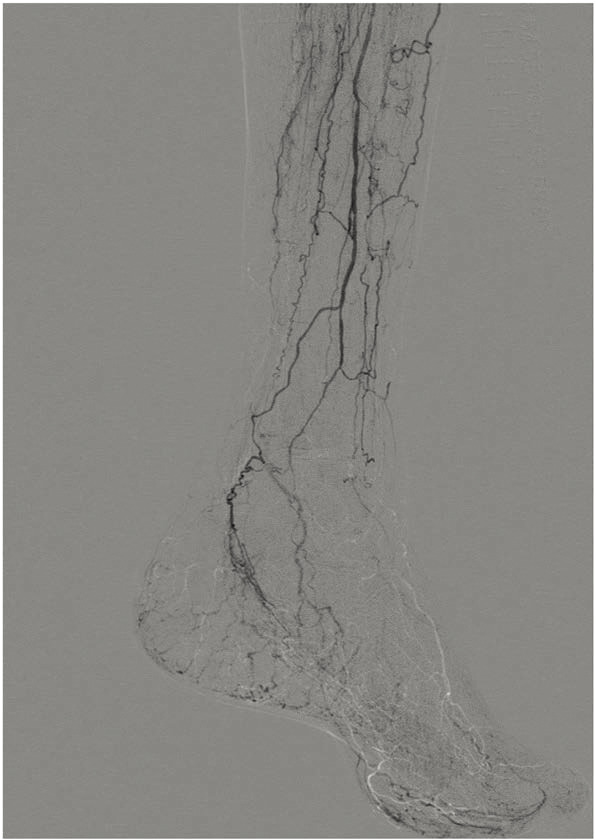
Fig. 3 Angiographic findings in diabetic patients with reduced peripheral vascular bed. The so-called “desert foot,” a condition in which the remaining peripheral vascular bed is small and all the main vessels are occluded, is not amenable to bypass or endovascular treatment and is difficult to salvage in severe cases. Early diabetic control is important.

Vascular lesions in patients with diabetes should be aggressively treated because they are directly linked to survival, QOL, and Activities of Daily Living (ADL); at the same time, various issues remain because they constitute a high-risk patient group. The issues that remain to be overcome in the future include risks associated with arteriosclerotic diseases in various areas, such as cardiovascular and cerebrovascular areas, as well as delayed wound healing, increased susceptibility to infection, arterial calcification, and reduced peripheral vascular bed of the legs due to diabetes (**Fig. 3**). Many such problems remain in diabetic feet and often complicate treatment; in particular, leg amputation immeasurably reduces patients’ survival, QOL, and ADL.^26)^ Vascular surgeons must not only perform revascularization but also use conservative treatment and new treatment methods, such as recent therapeutic angiogenesis, to save the limbs and lives of patients with diabetes as much as possible.

## Disclosure Statement

The author has no conflicts of interest to declare.

## Additional Remark

This paper was presented at the 34th educational seminar at the 50th Annual Meeting of the Japanese Society for Vascular Surgery (2022, Kitakyushu).
